# Dopaminergic Regulation of Circadian Food Anticipatory Activity Rhythms in the Rat

**DOI:** 10.1371/journal.pone.0082381

**Published:** 2013-11-29

**Authors:** Andrea N. Smit, Danica F. Patton, Mateusz Michalik, Hanna Opiol, Ralph E. Mistlberger

**Affiliations:** Department of Psychology, Simon Fraser University, Burnaby, BC, Canada; Kent State University, United States of America

## Abstract

Circadian activity rhythms are jointly controlled by a master pacemaker in the hypothalamic suprachiasmatic nuclei (SCN) and by food-entrainable circadian oscillators (FEOs) located elsewhere. The SCN mediates synchrony to daily light-dark cycles, whereas FEOs generate activity rhythms synchronized with regular daily mealtimes. The location of FEOs generating food anticipation rhythms, and the pathways that entrain these FEOs, remain to be clarified. To gain insight into entrainment pathways, we developed a protocol for measuring phase shifts of anticipatory activity rhythms in response to pharmacological probes. We used this protocol to examine a role for dopamine signaling in the timing of circadian food anticipation. To generate a stable food anticipation rhythm, rats were fed 3h/day beginning 6-h after lights-on or in constant light for at least 3 weeks. Rats then received the D2 agonist quinpirole (1 mg/kg IP) alone or after pretreatment with the dopamine synthesis inhibitor α-methylparatyrosine (AMPT). By comparison with vehicle injections, quinpirole administered 1-h before lights-off (19h before mealtime) induced a phase delay of activity onset prior to the next meal. Delay shifts were larger in rats pretreated with AMPT, and smaller following quinpirole administered 4-h after lights-on. A significant shift was not observed in response to the D1 agonist SKF81297. These results provide evidence that signaling at D2 receptors is involved in phase control of FEOs responsible for circadian food anticipatory rhythms in rats.

## Introduction

In mammals, daily rhythms of physiology and behavior are jointly controlled by a master light-entrainable circadian pacemaker in the suprachiasmatic nuclei (SCN) of the hypothalamus, and by food-entrainable oscillators (FEOs) located elsewhere. The SCN are critical for entrainment of daily rhythms to LD cycles, while FEOs are responsible for generating the daily rhythm of food anticipatory activity that emerges when rodents are restricted to one or two daily meals at a fixed time of day. Food anticipatory rhythms exhibit canonical properties of circadian clock control, but do not require the SCN pacemaker [1-3]. The location of FEOs driving food anticipatory activity is uncertain, and input pathways by which central FEOs are entrained have not yet been identified [4]. 

There are several reasons to suspect involvement of reward pathways in circadian food anticipation. Food is a strong reinforcer and activates central reward circuits [5]. Anticipatory activity rhythms can also be induced by daily schedules of access to other reward stimuli, including water [6], mates [7,8], drugs of abuse [9] and palatable food without caloric restriction [10-12]. These findings raise the possibility that neural correlates of reward can entrain circadian oscillators that drive food anticipatory rhythms [13].

Dopamine signaling in the ventral and dorsal striatum is critical for processing of reward stimuli and expression of motivated activity [14,15]. Both of these regions exhibit daily rhythms of circadian clock gene expression in rats and mice fed ad-libitum [16,17]. In both regions clock gene rhythms can be shifted by daily feeding schedules [17-19]. In the dorsal striatum clock gene rhythms can also be shifted in vivo by activation of dopamine D2 receptors, but not D1 receptors [20,21]. Unpredictable feeding schedules disrupt clock gene expression in the dorsal striatum [22] and dopamine antagonists reduce the magnitude of food anticipatory activity in rats and mice [23,24]. Lesions of the core of the nucleus accumbens (ventral striatum) have been reported to attenuate food anticipatory activity in rats [25], although ablation of both the core and shell of the nucleus accumbens does not [23]. Similar lesion studies have not been conducted on the dorsal striatum, where damage can be expected to impair locomotor output. Finally, chronic treatment with methamphetamine and other dopaminergic psychostimulant drugs can induce a circadian activity rhythm in otherwise arrhythmic, SCN-ablated rats and mice [18,26-30]. Methamphetamine-induced rhythmicity, like food restriction schedules, is associated with induction or shifting of circadian clock gene rhythms in the striatum but not the SCN [29]. Both methamphetamine and restricted feeding-induced rhythms exhibit an apparent shortening of intrinsic periodicity in a per1^-/-^/per2^-/-^/per3^-/-^ triple knockout mouse [30]. Rhythms induced by chronic methamphetamine and timed daily feeding may involve a common dopaminergic substrate.

Taken together, these results indicate that striatal clock genes are regulated by both food and dopamine. If striatal FEOs participate in food anticipatory activity rhythms, then these rhythms should be susceptible to phase shifting by dopaminergic stimuli. To test this hypothesis, we developed a protocol for measuring phase shifts of food anticipatory rhythms by pharmacological probes. We report here evidence consistent with regulation of food anticipatory circadian rhythms by dopamine D2 receptor signaling in the dorsal striatum.

## Materials and Methods

### Subjects and apparatus

Male Sprague Dawley Rats (Charles River, Quebec) were housed singly in standard plastic cages equipped with a running wheel or overhead motion sensor. Cages were housed in a sound attenuated, climate controlled vivarium with a 12:12 light/dark (LD) cycle. Activity data were monitored continuously by computer using the ClockLab (Actimetrics) data acquisition system. All procedures were approved by the University Animal Care Committee at Simon Fraser University (permit #935p).

### Experiment 1: Effect of the D2 agonist quinpirole on food anticipatory activity onset

The objective of the first experiment was to determine whether an acute injection of the D2 agonist quinpirole can shift the onset of food anticipatory activity on the following day. Rats (N=16) in wheel running cages were maintained on ad-lib access to food and water for 2 weeks. The rats were food deprived overnight, and then provided food for 3-h each day, beginning 6-h after lights on (Zeitgeber Time 6, where ZT0 is lights on, by convention). On day 21 of restricted feeding, at ZT11 (1-h before lights-off), 8 rats received 1 mg/kg IP of quinpirole hydrochloride (Sigma-Aldrich), dissolved in sterile water at a concentration of 1mg/ml. Quinpirole at this dose and time of day has been shown to suppress clock gene expression in the dorsal striatum, measured the following day early in the light period [20]. The other 8 rats received sterile water alone. The next day, the lights remained off and the rats were not fed. The daily feeding and LD schedules then resumed for 5 days, and the procedure was repeated, with the groups reversed. This counterbalanced sequence was repeated, with injections made at ZT4. Lights were turned off after the ZT4 injections, and remained off with no food until the next day at scheduled mealtime, when the feeding and LD schedules were resumed. In the ZT4 injection condition, for the first meal after the food deprivation day, the rats were given a limited amount of food rather than a limited duration of food access as on all other days. The dependent variable was the time at which food anticipatory activity began on the day after the injections, relative to anticipatory activity onset times averaged over the 4-5 preceding days of restricted feeding. This difference in timing was then compared across conditions (drug vs. vehicle) to arrive at the phase shift attributable to drug. 

### Experiment 2: Effect of quinpirole following acute dopamine synthesis inhibition

The objective of the second experiment was to determine whether suppression of dopamine synthesis 10h prior to a quinpirole injection would induce D2 receptor supersensitivity sufficient to potentiate the size of phase shifts of food anticipatory activity. Rats (N=14) in wheel running cages were restricted to a 3-h daily meal (ZT6-9) for 18 days, which was reduced to 2-h (ZT6-8) for another 50 days. The rats then received the tyrosine hydroxylase inhibitor D,L-alpha-methyl-paratyrosine HCL (AMPT; 200mg/kg in sterile water, IP) at ZT1, followed by either quinpirole (1mg/kg; N=7 rats) or vehicle injection (N=7 rats) at ZT11. The rats were food deprived the next day. Two weeks later the procedure was repeated, with the rats receiving a vehicle injection at both ZT1 and ZT11. 

### Experiment 3: Effect of quinpirole on food anticipatory activity in rats maintained in constant light

The objective of the third experiment was to determine whether phase shifts of food anticipatory activity onset induced by quinpirole were independent of LD-entrained rhythms controlled by the SCN pacemaker. To severely dampen or eliminate SCN controlled rhythms, rats (N=18) were maintained in constant light (LL, ~300 lux) for 21 days. Instead of running wheels, overhead passive infra-red motion sensors were used to measure activity because LL suppresses running, reducing the precision of activity onsets and increasing measurement error. The rats were then restricted to a 3-h daily meal for 63 days. Mealtime was delay shifted by 8h on day 21 for reasons unrelated to the objectives of this study. On day 42 of food restriction, 2h after the end of mealtime (matching the ZT11 injection time in Experiments 1 and 2, relative to mealtime) the rats received an injection of quinpirole (N=6), vehicle (N=6) or no injection (N=6). All rats were food deprived the next day, after which the feeding schedule was resumed. This sequence was repeated twice more at 7 day intervals, to complete a counterbalanced within-subjects design. 

### Experiment 4: Effect of the D1 agonist SKF81297 on food anticipatory activity

The objective of the last experiment was to determine whether food anticipatory activity could be phase shifted by selective activation of the D1 receptor. Rats (N=16) in running wheels in LD received food for 3-h/day at ZT6-9 for 21 days, and then received an injection of the D1 agonist SKF 81297 (Sigma-Aldrich; 1 mg/kg IP) (N=6) or vehicle (N=5) at ZT11, or no injection (N=5). The dose was chosen based on prior work demonstrating effects on striatal neurons and behavior [20,31]. The rats were food deprived in constant dark the next day after which the feeding schedule was resumed in LD. This sequence was repeated once more 7 days later, yielding within-subject contrasts between SKF81297 vs. vehicle (N=6), SKF81297 vs. no injection (N=5) and vehicle vs. no injection (N=5). 

### Data analyses

For wheel running data, the daily onset of food anticipatory activity was identified using the Clocklab (Actimetrics) algorithm. For motion sensor data (Experiment 3, conducted in LL), onsets were defined as the first 5 min bin within 3-h of mealtime when activity counts exceeded a threshold of 50% of the maximum level of activity prior to mealtime, and remained above 30% of the maximum in at least 50% of the next 12 time bins (1h). To quantify phase shifts, activity onset on the day after injections was compared with the average pre-meal activity onset during the 4-5 days immediately preceding that injection. Drug, vehicle and no injection conditions were then compared using ANOVA and paired or independent t-tests. 

## Results

### Experiment 1: D2 agonist quinpirole phase shifts food anticipatory activity onset

**Figure 1:**
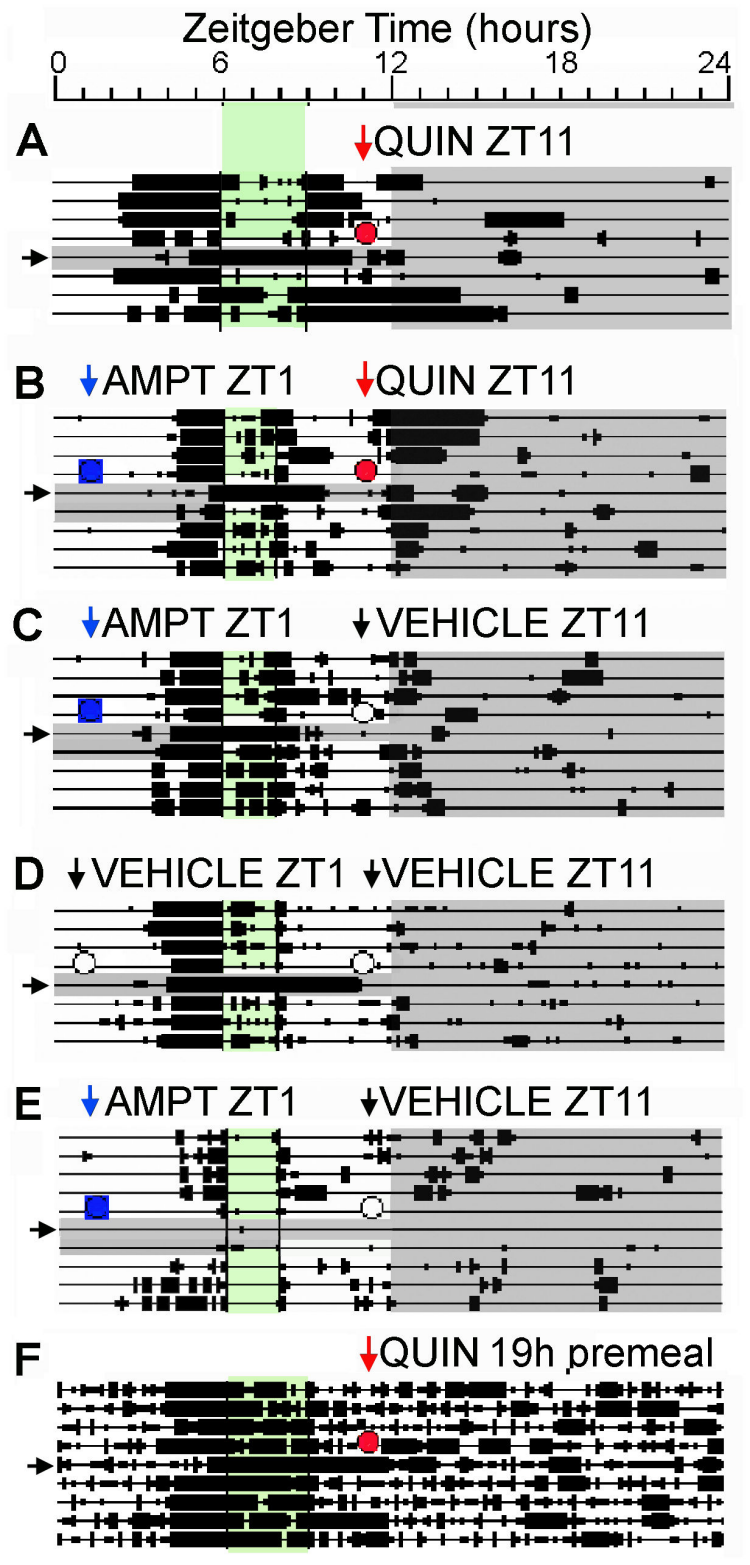
Activity of rats during restricted feeding, and effects of drug and vehicle injections. Each line represents 24h of recording, with time in 10 min bins plotted from left to right, and consecutive days aligned vertically. Time bins during which 1 or more wheel revolutions (A-E) or general activity counts (F) were registered are represented by heavy bars, the height of which corresponds to activity level (in quartiles). Mealtime occurred between hours 6-9 (Panels A, F) or 6-8 (Panels B-E) and is shaded green. Injections are denoted by red circles and arrows for quinpirole (QUIN, 1 mg/kg), blue squares and arrows for alpha-methylparatyrosine (AMPT, 200 mg/kg), and unfilled circles and black arrows for vehicle (sterile water). Grey shading indicates lights-off, as part of the daily LD cycle or the 1 or 2 days following drug injections. Food anticipatory activity onset was measured on the 4 days prior to injections and on the day after injections. Food anticipatory activity onset was delayed on the day following quinpirole (A,F) or quinpirole with AMPT pretreatment (B), but was not delayed on the day following AMPT (C) or vehicle alone (D). Some rats stopped using the running wheels for 2 or more days after AMPT and could not be used to measure phase shifts (e.g., E).

**Figure 2:**
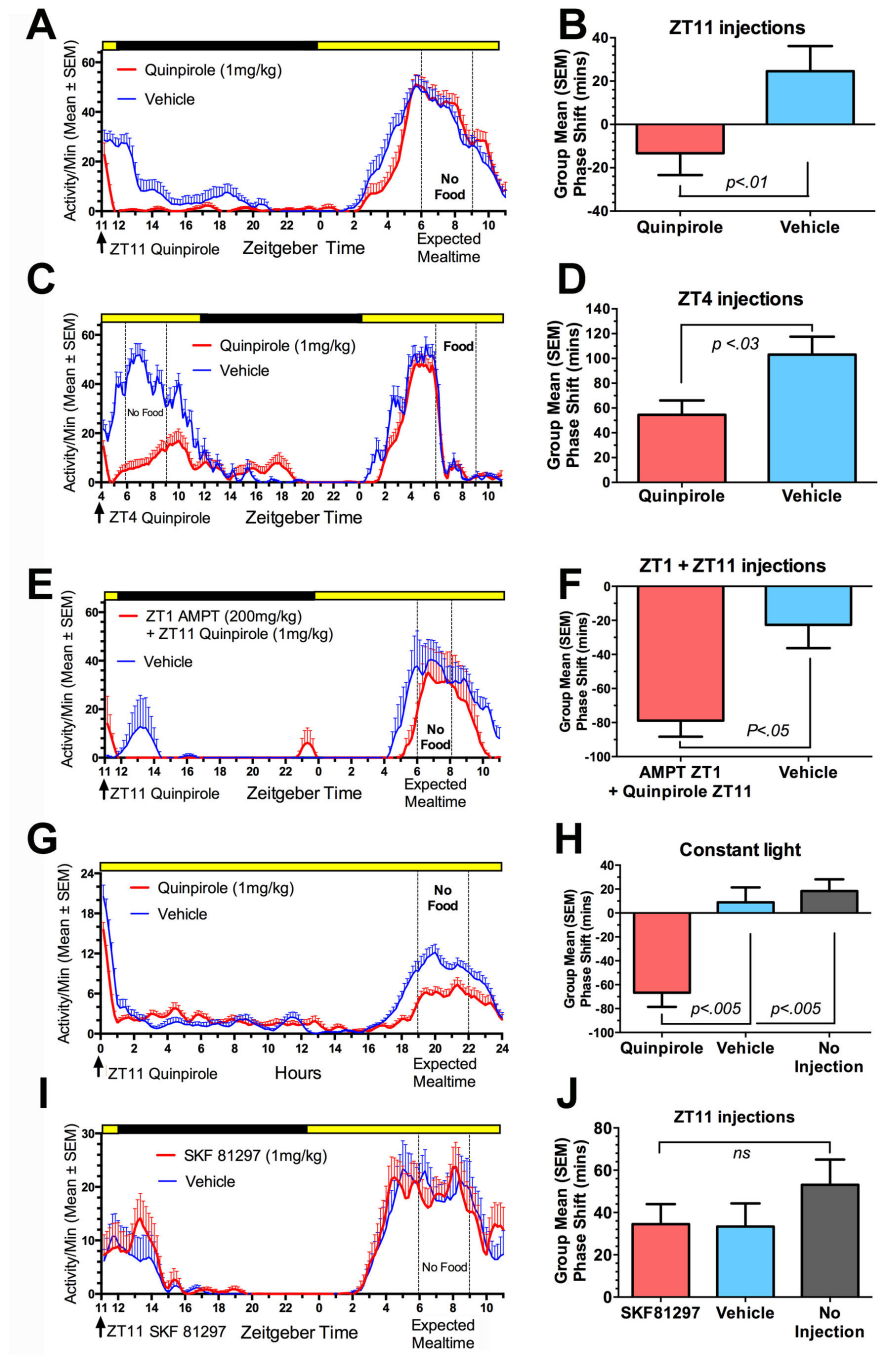
Group mean (+ SEM) average waveforms of activity during restricted feeding on the day following injections of drug (red curves) or vehicle (blue curves) in Experiments 1-4. Injection times (in zeitgeber time, where ZT0 is lights-on by convention) are indicated by the arrows and scheduled mealtimes by the vertical dotted lines. The LD cycle is indicated by the black and yellow bars (A,C,E,I); note, however, that in those experiments, the lights were kept off during the days represented here (i.e., the day after injection). The bar graphs represent phase shifts of premeal activity onset on the day after the injections by comparison with the 4 days prior to injection. A delay in the onset of premeal activity is represented by negative values, and an advance by positive values. Note that quinpirole injections induced delays relative to vehicle injections in all experiments. P-values are the results of paired t-tests.

**Figure 3:**
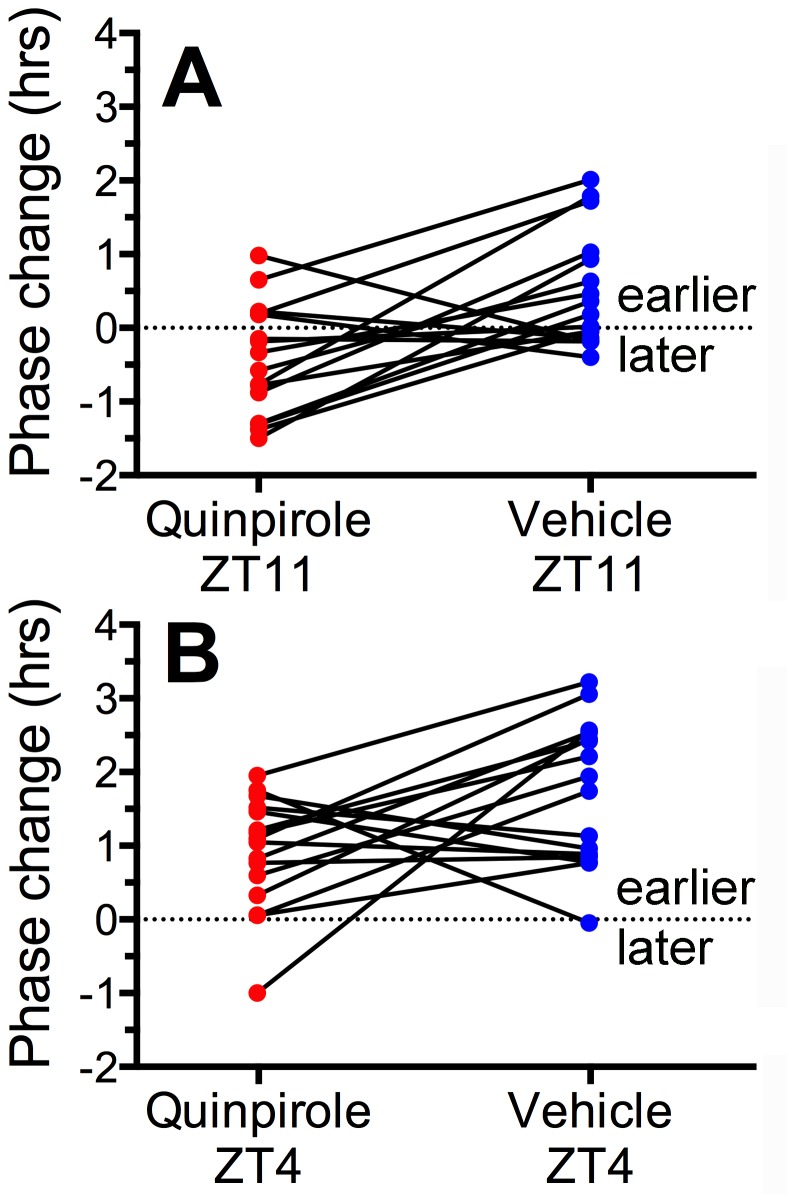
Changes in the timing of food anticipatory activity following quinpirole (1 mg/kg; red dots) or vehicle injections (blue dots) administered on the previous day at ZT11 (1h before lights off) or ZT4 (4h after lights-on) in Experiment 1. Each data point represents the difference in hours between food anticipatory activity onset on the day following injections compared to food anticipatory activity onset averaged over the 4 days preceding the injections. Positive values indicate phase advances (earlier onset of activity) and negative values indicate phase delays (later onset of activity). Within-subject pairs are connected by lines, illustrating that in most rats, activity onsets were delayed following quinpirole injections relative to vehicle injections.

During the 5 days of restricted feeding preceding the first set of injections, food anticipatory activity began on average 2.42 + .5 h (group mean + SD) prior to mealtime (Figures 1,2). Relative to this baseline, most rats exhibited a delay of activity onset on the day following quinpirole at ZT11 and an advance on the day following quinpirole at ZT4 (Figure 3a, b). However, relative to the vehicle injections, quinpirole induced a phase delay of food anticipatory activity onset in 12 of 16 cases at ZT11 (mean difference = -.94 + 1.1 h, paired *t*_*(15)*_= 3.51, p= .003; Figures 2a,b, 3a) and in 11 of 16 cases at ZT4 (mean difference = -.81 + 1.35 h, paired *t*_*(15)*_ =2.61, p=.014; Figures 2c,d, 3b). 

Quinpirole injections at both time points acutely reduced wheel running activity for ~6-8h. Consequently, in the ZT4 injection condition, the rats were not fed on the day of the injection and food anticipatory onset was measured the next day. Food anticipation onset was advanced (occurred earlier) in both the vehicle and the drug condition, compared to the immediately preceding days and to the ZT11 injection condition, but this is attributable to increased hunger due to food depriving the rats on the day of the injection. Despite the advance of activity on both vehicle and drug groups in the ZT4 injection condition, the drug group was significantly phase delayed relative to the vehicle group.

### Experiment 2: Quinpirole-induced phase shifts are potentiated by DA synthesis inhibition

Preliminary tests identified 200 mg/kg AMPT as a dose that did not suppress wheel running activity the next day. During restricted feeding, administration of this dose at ZT1 caused an unexpected severe hypokinesia in 9 of 14 rats (e.g., Figure 1e), reducing the sample size to 5. In this subgroup, quinpirole (1 mg/kg) at ZT11 following AMPT at ZT1 significantly delayed FAA onset the next day by comparison with the vehicle-vehicle treatment (-1.47 + .07 h vs -.39 + .41 h, paired *t*_*2*_ =4.23, p=.05; Figures 1b, 2e,f). This delay shift was 61% larger than the average observed to quinpirole alone in Experiment 1 (1-tailed unpaired *t*_*(16)*_ =1.95, p=.034). The delay shift of food anticipatory activity onset in the AMPT-quinpirole condition was also significant by contrast with the AMPT-vehicle condition (.58 + .07h, *t*_*(2)*_ = 25.7, p=.004). The AMPT-vehicle treatment produced a small phase advance relative to the vehicle-vehicle treatment (-.38 + .22, *t*_*(2)*_= 3.93, p=.046). In the AMPT-quinpirole condition, food anticipatory activity rose to the same peak level, but ended earlier compared to the vehicle-vehicle condition (Figure 2e).

### Experiment 3: Quinpirole phase shifts food anticipatory activity in rats maintained in LL

Exposure to LL for 3 weeks resulted in severe damping or loss of free-running activity rhythms when food was available ad-libitum. When food was restricted to a 3-h daily meal, food anticipatory activity emerged within a few days. Food anticipatory activity in LL rats was more variable in onset and lower in magnitude relative to LD rats, but a quantitative comparison of the two conditions is precluded due to the use of different activity measures (motion sensors Vs. running wheels, respectively). 

Quinpirole (1 mg/kg) administered 19h prior to the next mealtime in LL (comparable to ZT11 in LD) significantly delayed food anticipatory activity onset (F_(2,28)_ = 11.9, p=.002) relative to vehicle (p=.004) and no injection (p=.0002) (Figures 1f, 2g-h). Food anticipatory activity onset did not differ following vehicle injections by comparison with no injections. Quinpirole, compared to vehicle, suppressed wheel running for only ~1h post-injection, indicating that phase delays of food anticipatory activity onset the day after quinpirole injections does not require the longer bouts of reduced wheel running evident in rats maintained in LD.

### Experiment 4: D1 agonist SKF81297 does not phase shift food anticipatory activity

The D1 agonist SKF81297 administered at ZT11 did not alter food anticipatory activity onset by contrast with a vehicle injection (*t*_*(5)*_= 0.35, p=.70) or with no injection (*t*_*(4)*_ = 0.23, p=.82). There was also no significant difference in FAA timing between the vehicle injection and no-injection conditions (Figure 2i,j).

## Discussion

In a variety of species, restricting food access to a fixed time of day induces a daily rhythm of food anticipatory activity. In neurologically intact and SCN-ablated rats and mice, the formal properties of these behavioral rhythms are consistent with control by food-entrainable circadian oscillators located outside of the SCN circadian pacemaker [2,3,32]. Food-entrainable oscillations of circadian clock gene expression and other cellular activities have been observed in a variety of other brain regions and peripheral tissues, but lesion approaches have yet to reveal a critical role for any of these regions [3,4,32,33]. Stimuli critical for phase control of non-SCN circadian oscillators by food have also not been established. In the present study, we developed a complementary approach to localizing oscillators and entrainment pathways for food anticipatory rhythms using pharmacological probes and procedures for measuring shifts in the timing of food anticipation. Similar methodology has been instrumental in characterizing neurochemical input pathways by which photic and nonphotic stimuli can shift and entrain the SCN pacemaker. 

With this approach we observed that activation of dopamine D2 receptors, but not D1 receptors, can shift the onset of food anticipatory activity in rats maintained in LD or LL, and that this effect can be potentiated by prior treatment with a dopamine synthesis inhibitor. Mesolimbic and nigrostriatal dopamine pathways are critical for processing of reward stimuli and for expression of motivated behavior, of which food anticipatory activity is an exemplar. Consequently, it is plausible to expect that the expression of food anticipatory activity will depend on dopamine signaling in one or more ways. Circadian clock genes exhibit daily rhythms of expression in the dorsal striatum that can be entrained by scheduled feeding (e.g. [17,18]), and acutely downregulated by quinpirole [20,21]. This suggests a hypothesis that dopamine sensitive circadian oscillators in the dorsal striatum may regulate the timing of daily activity rhythms entrained to feeding time. Using the Hood et al [20] study as a guide to injection times and doses, we obtained novel behavioral and clock gene evidence consistent with this hypothesis. The sensitivity of activity rhythms and dorsal striatal clock gene rhythms in vivo to a D2 agonist but not a D1 agonist supports a functional relationship between these rhythms. A role for dopamine-sensitive circadian oscillators in other structures is of course not ruled out, although lesion studies have revealed essentially normal food anticipatory activity rhythms in rats lacking a neocortex or a ventral striatum [3,23]. 

A critical interpretive issue is whether the delayed onset of food anticipatory activity observed on the day following quinpirole administration reflects a shift in phase of a circadian oscillator, a change in its amplitude, or a change in a threshold for induction of premeal activity by a mechanism independent of a circadian timer. The results of Experiment 1 fit more closely with a phase shift interpretation because in the drug condition, the premeal rise of activity begins later but peaks at the same level as in the vehicle injection condition, indicating no oscillator damping or threshold change. The falling phase of the food anticipation rhythm showed only a trend for a delay, but this portion of the activity waveform is more difficult to interpret, given evidence that it is also regulated by an interval timer that measures the expected duration of meal access independent of its onset [34]. The onset of food anticipatory activity to meals scheduled during the light period exhibits properties of circadian timing but not interval timing [35], so the rising and falling phases of anticipatory rhythms could be differentially affected by drug treatments. The rising and falling phases may also be regulated by coupling within a population of FEOs, which could be differentially shifted by a discrete drug stimulus. Evidence for multiple oscillators underlying food anticipatory activity is available from studies of single and multiple daily meal schedules [32,35,36]. A dual oscillator model of the SCN pacemaker can account for differential shifting of the onset and end of the daily active phase in ad-libitum fed rodents exposed to a discrete light pulse in constant dark [37]. 

In rats pretreated with AMPT or maintained in LL, the onset of food anticipatory activity was similarly delayed by quinpirole, but other features of the activity waveform were also altered. In AMPT treated rats, the waveform was compressed, while in LL, the waveform peak was reduced. Again these changes could reflect differential regulation of food anticipatory activity onset, duration and amplitude by dopaminergic compounds interacting with lighting conditions. Additional work will be needed to resolve the role of dopamine signaling in the onset, duration and amplitude dimensions of food anticipatory rhythms.

Dopaminergic involvement in food anticipatory activity has previously been suggested based on evidence that the total amount of activity prior to mealtime is reduced following acute treatment with a nonspecific dopamine antagonist [23], or with D1 and D2 specific antagonists [24]. Notably, the maximum suppression using D1 and D2 antagonists in combination was only ~50% [24]. These experiments did not measure the timing of food anticipatory activity, and thus do not provide evidence that the antagonists have directly affected a food-entrainable oscillator. Alterations in the amount of food anticipatory activity following drug treatment could be mediated by actions entirely downstream from the clock mechanism. Evidence that a drug affects a circadian clock requires demonstration that a property of the clock, such as its phase or period, has been affected. Changes in the amount of activity are ambiguous without a concurrent measure of rhythm timing. These considerations also apply to the interpretation of lesion and gene knockout studies of food anticipatory rhythms.

Experimental manipulations of feeding schedules have revealed the existence of a circadian oscillator system in mammals specialized for guiding food seeking behavior in the circadian domain. This system presumably plays an important role in optimal foraging when food availability varies predictably with time of day. Evidence that dopaminergic reward pathways may entrain circadian oscillators that drive daily rhythms of food seeking activity raises the possibility that rewards generally, including drugs of abuse used on a circadian schedule (as many are), may recruit these oscillators, creating circadian vulnerabilities to drug taking and relapse. 
